# Supplementary material for: Canonical WNT signalling governs Echinococcus metacestode development
Source: PLoS Pathog. 2026 Mar 23;22(3):e1014046. doi: 10.1371/journal.ppat.1014046 (PMC13029709; doi:10.1371/journal.ppat.1014046)
Supplement: S1 Fig — (PDF) [file ppat.1014046.s001.pdf]

S1 Figure

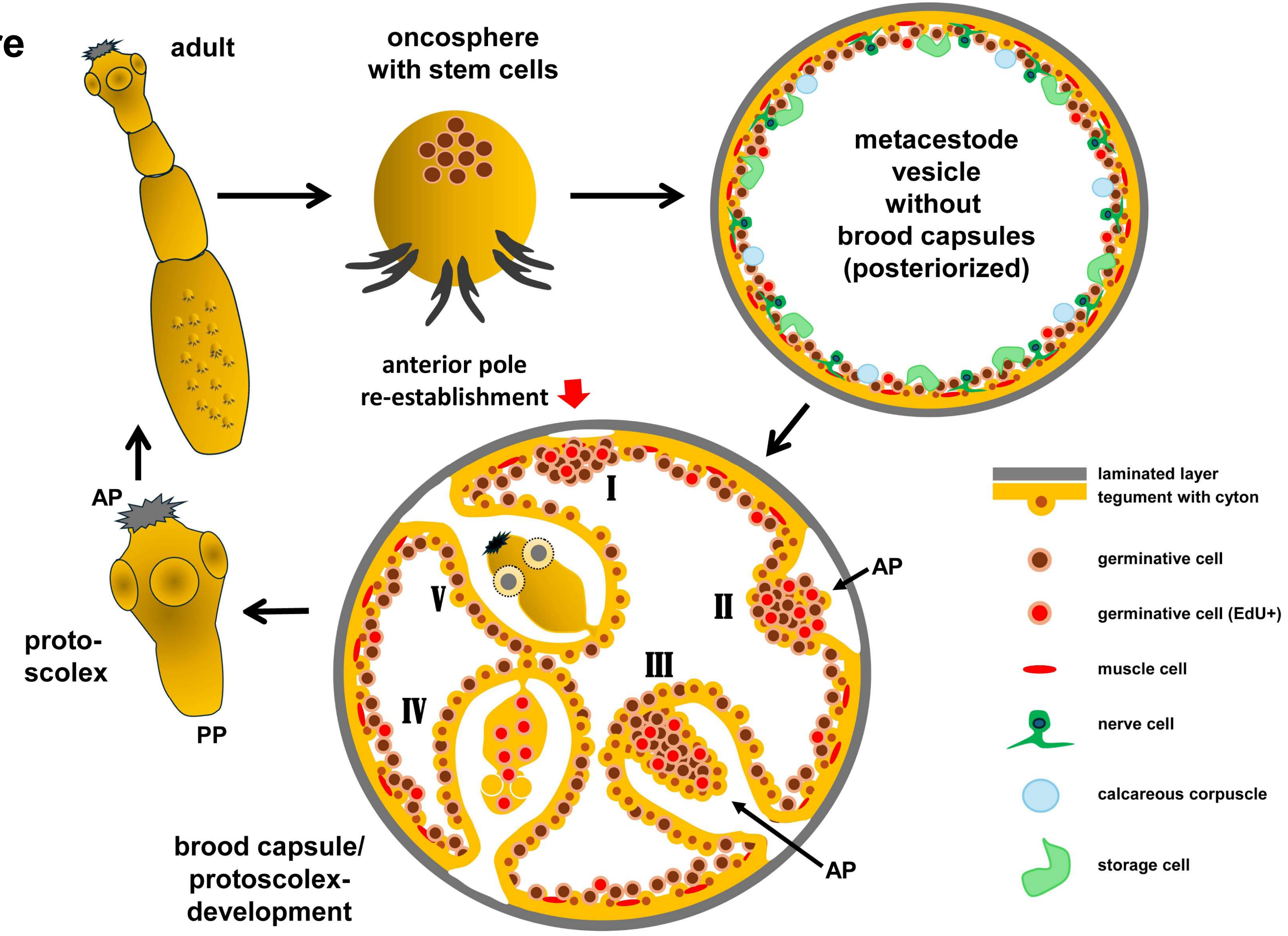

S1 Figure. Schematic illustration of *E. multilocularis* life-cycle stages. Shown are images of the adult worm that produces infective eggs which contain the oncosphere. To the intermediate host the oncosphere delivers germinative cells that produce the metacestode. The metacestode (consisting of laminated layer, tegument, germinative-, muscle-, nerve-, and storage cells) grows infiltratively as fully posteriorized tissue until the anterior pole is re-established in the form of young brood capsules (stage I). Brood capsule and protoscolex development involves stem cell proliferation and differentiation through stages II – IV. In the fully developed protoscolex (stage V; with anterior and posterior pole), germinative cell proliferation ceases until the tissue is passaged through the stomach of the definitive host. A legend indicated the different cell types is shown to the right. AP and PP indicate anterior and posterior pole, respectively. Consecutive stages of brood capsule/protoscolex development are indicated by I – V. For better visualization, the protoscolex is exclusively shown in evaginated form and nerve-/storage cells are omitted in brood capsule development image. For references, please refer to the manuscript text.
