# Supplementary material for: Canonical WNT signalling governs Echinococcus metacestode development
Source: PLoS Pathog. 2026 Mar 23;22(3):e1014046. doi: 10.1371/journal.ppat.1014046 (PMC13029709; doi:10.1371/journal.ppat.1014046)
Supplement: S2 Fig — (PDF) [file ppat.1014046.s002.pdf]

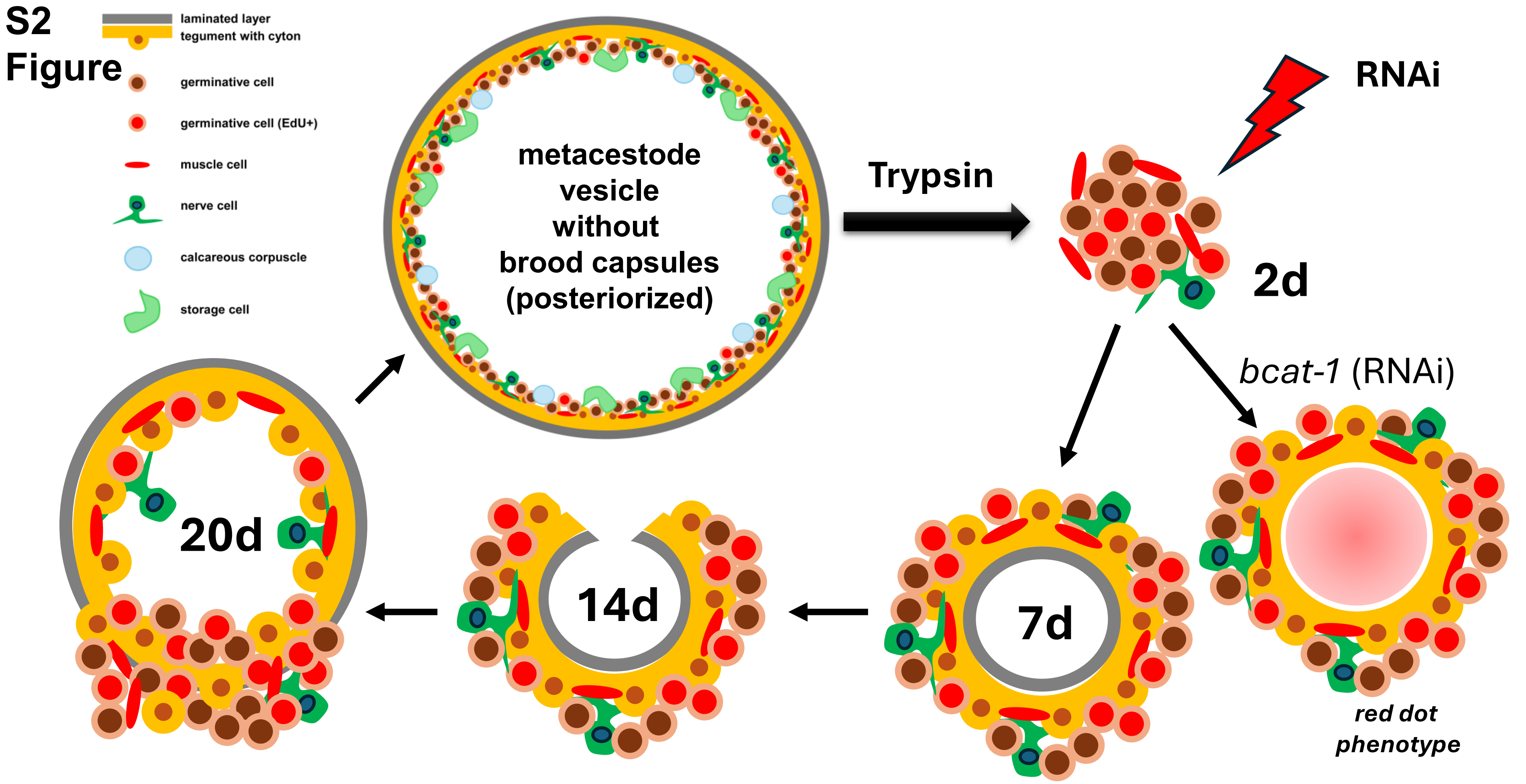

S2 Figure. Schematic illustration of *E. multilocularis* primary cell cultivation system. *In vitro* cultivated metacestode vesicles are trypsin-digested and seeded under axenic (anaerobic) growth conditions. After 2 days of culture, aggregates form that mostly (~85%) contain germinative cells but also (*wnt1*+) muscle and nerve cells. Under the influence of posteriorizing muscle cells, germinative cells proliferate and differentiate towards posterior cell types. After ~ 7 days, a metacestode tegument is formed which secretes a laminated layer. These aggregates are arranged as inside-out vesicles. After ~ 14 days of development lesions in cell and laminated layer lead to inversion of the orientation (snap) and after ~ 20 days of culture, fully mature metacestode vesicles emerge from cell aggregates. At 2 days of incubation, RNAi is routinely applied to cell cultures (as indicated). In the case of *bcat-1*(RNAi), red-dot aggregates are formed that lack a laminated layer and presumable accumulate phenol red. For microscopic images supporting these consecutive steps of primary cell development, please see references [2,18,23] of the main text. A legend explaining the structures and cell types is shown to the left.
