# Supplementary material for: Canonical WNT signalling governs Echinococcus metacestode development
Source: PLoS Pathog. 2026 Mar 23;22(3):e1014046. doi: 10.1371/journal.ppat.1014046 (PMC13029709; doi:10.1371/journal.ppat.1014046)
Supplement: S3 Fig — (PDF) [file ppat.1014046.s003.pdf]

S3 Figure

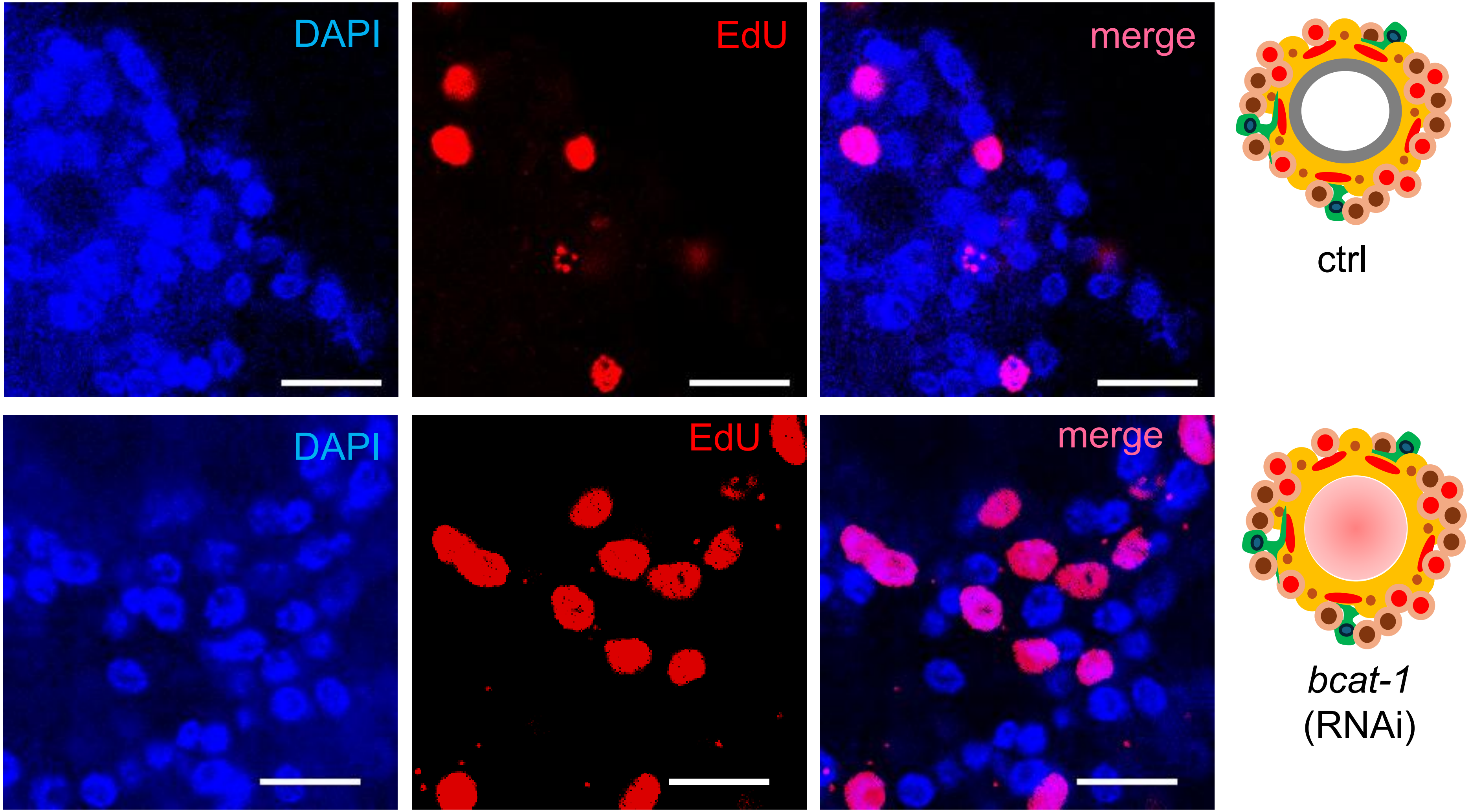

S3 Figure. Hyperproliferation of germinative cells in primary cell aggregates after *bcat-1*(RNAi). Shown are single confocal slices of primary cell aggregates in control (ctrl; upper panel) and *bcat-1*(RNAi) (lower panel) cultures (siRNA approach). Channels are blue (DAPI, nuclei), red (EdU, prolifertating stem cells) and merge of both channels (as indicated). Size bars indicate 10 µm in all images. Images to the right represent parasite primary cell cultures of control (ctrl) and *bcat-1*(RNAi) as detailed in S2 Figure.
