# Supplementary material for: Canonical WNT signalling governs Echinococcus metacestode development
Source: PLoS Pathog. 2026 Mar 23;22(3):e1014046. doi: 10.1371/journal.ppat.1014046 (PMC13029709; doi:10.1371/journal.ppat.1014046)
Supplement: S4 Fig — (PDF) [file ppat.1014046.s004.pdf]

S4 Figure

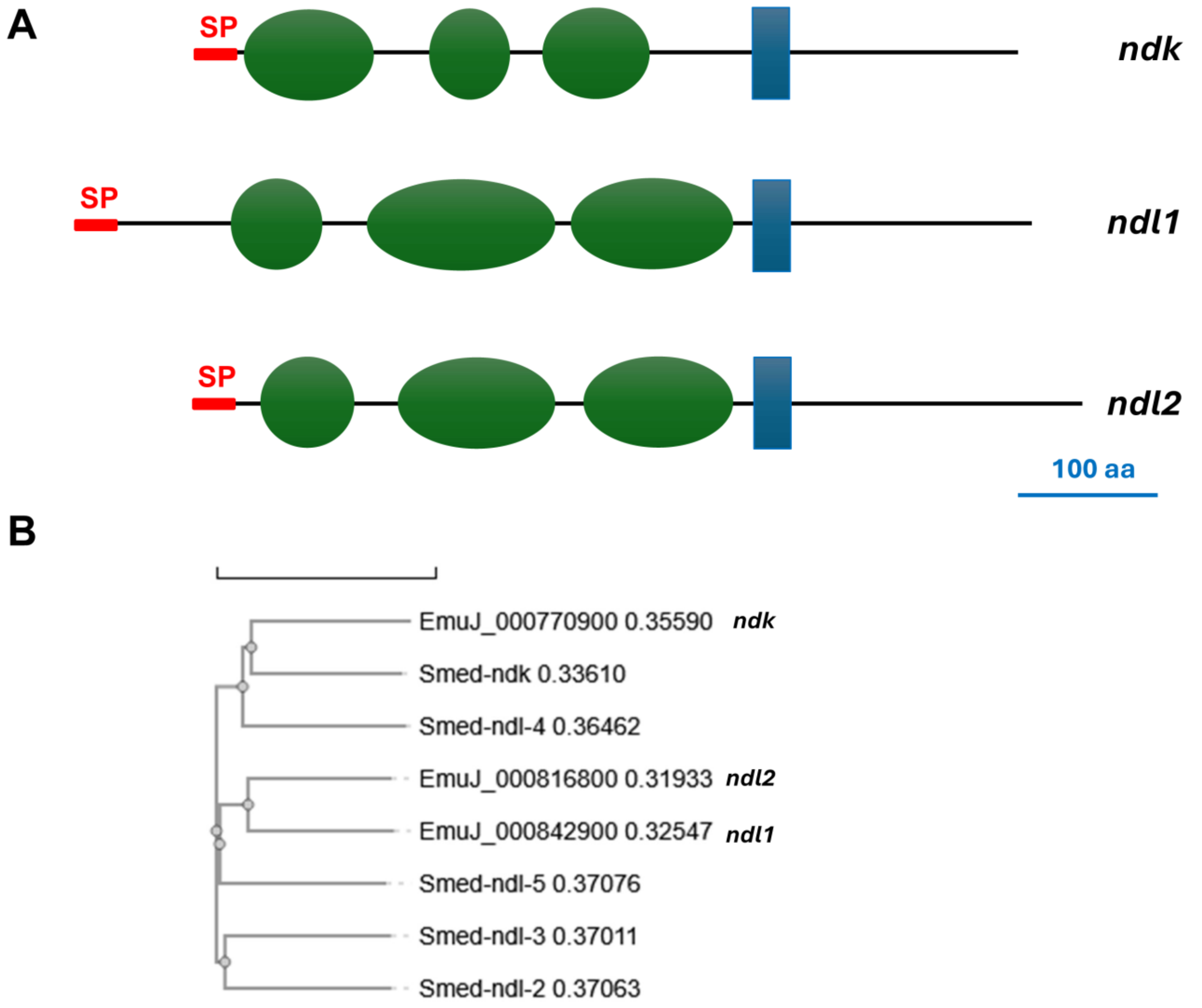

S4 Figure. Structure and homologies of *Echinococcus ndk* and *ndl* (*ndk*-like) factors. (A) Domain structure of *E. multilocularis ndk*, *ndl1*, and *ndl2* (as indicated). Shown are transmembrane domains (blue), Ig-Domains (green), and signal peptides (red). Please note that all three proteins display the characteristic 3 extracellular Ig-domains, but no intracellular kinase domain (as in FGF receptors). Size bar indicates 100 amino acids. (B) Phylogenetic analysis of *Echinococcus ndk*, *ndl1*, and *ndl2* with *Schmidtea mediterranea ndk* and *ndl's*. Depicted are the *Echinococcus* factors with gene ID and name as well as all *Schmidtea ndk*-like factors.
