# Supplementary material for: Canonical WNT signalling governs Echinococcus metacestode development
Source: PLoS Pathog. 2026 Mar 23;22(3):e1014046. doi: 10.1371/journal.ppat.1014046 (PMC13029709; doi:10.1371/journal.ppat.1014046)
Supplement: S5 Fig — (PDF) [file ppat.1014046.s005.pdf]

S5 Figure

A

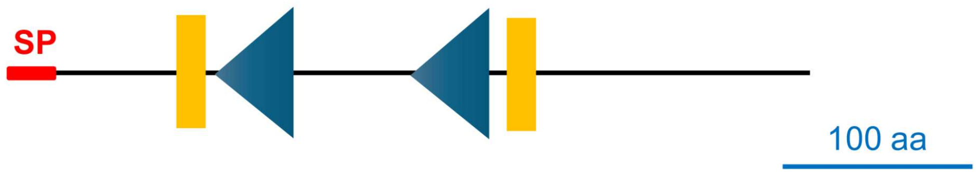

B

```
FST_HUMAN      MVRARHQPGGLCLLLLLLCQFMEDRSAQAGNCWLRQAKNGRCQVLY-KTELSKEECCSTG      59
FST_ECHMU      ---------MVPLLIFVLQALLVFTVSDAGSCFRIRLPNGRCHELLQRNYITRSECCRLG      51
FST_SCHMA      ---------------------------------------------MEESISQLECCKRG      14
                                                              .  ::: ***  *

FST_HUMAN      RLSTSWTEEDVNDNTLFKWMIFNGGAPNCIPC------KETCENVDCGPGKKCRMNKKNK      113
FST_ECHMU      GFYVSGHL--TEPQFVRDLLLRRTGIANCHYSCQRGFHNESCARVNCPDGFRCEVQN-HE      108
FST_SCHMA      GFYLNRQI--TQQEYLTDHLLFESGTPNCVQACNEELP-STCKGYVCPEGHYCRMIN-NE      70
                :  .     .: : : . :: . *  **  .       .:*    *  *  *.: : ::

FST_HUMAN      PRCVCAPDCSNITW-KGPVCGLDGKTYRNECALLKARCKEQPELEVQYQGR-----C---      164
FST_ECHMU      AVCACSSICIVEDFESGPVCTTDFRQFRNRCSFLKERCRNLDTLLVEMECPPADHVCPYR      168
FST_SCHMA      PKCKCRLQCNTADYLTGPLCTTNLRRFHNQCHLKQARCKSPKESLEVIPCPDEGLRCSDL      130
                 * *   *    : .**:*  : : ::*.* : : **:.                *

FST_HUMAN      ------------------------------------------------------------      164
FST_ECHMU      QNTS--------------------------------------------------------      172
FST_SCHMA      SILSEKNLNLNSLFKTLYAEYEERKGVPSLPSSSSLPSSTSTTKSSLSSTDNQEYTDNED      190

FST_HUMAN      -----------------------------------KKTCRDVFCPGSSTCVVDQTNNA-Y      188
FST_ECHMU      ------------------------HLE------DHDYAYPVRICPPNKVCIVRQYSGRAS      202
FST_SCHMA      DNVIIMNRRTSTDLKTYIDKMNDESIFDEKSPYSNDELSKELICNANEYCLLRQFDGRPS      250
                                                  .      :*  .. *:: * ..

FST_HUMAN      CVT-------------------CNRICPEPASSEQYLCGNDGVTYSSACHLRKATCLLGR      229
FST_ECHMU      CEDPNWLGRKHIESAFDRVAVSNLDSCANS-PYGSPICGTDNNTYNNQCHLRKASIQKGL      261
FST_SCHMA      CEYWN------------EQSWQTLNNCPES-TFNEPICSTDNQTFYNSCDLKLTGLKNQL      297
               *                         * :     . :*..*. *: . *.*: :

FST_HUMAN      SIGLAYEGKCIKAKSCEDIQCTGGK-KCLWDFKVGRGRCSLCDELCPDSKSDEPVCASDN      288
FST_ECHMU      EIRIAYLGPCRKNATCDQIRCQSPHMTCVPHVRTGQPVCLDCTDLPPNCNPRVFVRINPA      321
FST_SCHMA      EVRIAYKGECRSKVTCSDIQCPRPDMRCSPHHLTGQPICMDCAKLPLDCNASFRNGQETL      357
               .: :** * * .  :*.:*:*   .  *  .  .*:  *  * .*  :.:       .

FST_HUMAN      ATYASECAMKEAACSSGVLLEVKHSGSCNSISEDTEEEEEDEDQDYSFPISSILEW----      344
FST_ECHMU      EI---------AEMTLFEMFKEAKQ-----NF---GDPRWHGDVFTXWLASGMWKWKKVI      364
FST_SCHMA      TSSTSSSSSSSSSTGVLGKRKTQ-----IF---GDPVWKTPVQTY-------GWPVVC      402
                          :  :   : :   .           :   .              *

FST_HUMAN      ------------------------------------------------------------      344
FST_ECHMU      PQCLLPTRLQLYRQTLRRPCLNWILSSRVISELSIVR----------PQEQLKRYFILKK      414
FST_SCHMA      GS---------NGKTYRNTCFLHVVNCLSDKFVGLKKPEFCSETIPSKHDDWRRHFGK--      451

FST_HUMAN      ----------------------------------------   344
FST_ECHMU      ERTSEVKSITTIITTTFVTLTLASPHCCLSCALMHATQDP   454
FST_SCHMA      --------YSNL----F-----------------------   456
```

S5 Figure. Structure and homologies of *Echinococcus fst*. (A) Domain structure of *E. multilocularis* FST. Shown are characteristic follistatin N-terminal domains (FOLN, yellow), Kazal-type serine protease inhibitor domains (blue), and signal peptide (red). Size bar indicates 100 amino acids. (B) Amino acid sequence comparison betwen *E. multilocularis* FST (ECHMU), human FST (HUMAN), and *Schmidtea mediterranea* FST (SCHMA). Sites of perfect alignment (*) as well as groups of strong (:) or weak (.) similarity are marked below the sequences.
