# Supplementary material for: Canonical WNT signalling governs Echinococcus metacestode development
Source: PLoS Pathog. 2026 Mar 23;22(3):e1014046. doi: 10.1371/journal.ppat.1014046 (PMC13029709; doi:10.1371/journal.ppat.1014046)
Supplement: S6 Fig — (PDF) [file ppat.1014046.s006.pdf]

S6 Figure

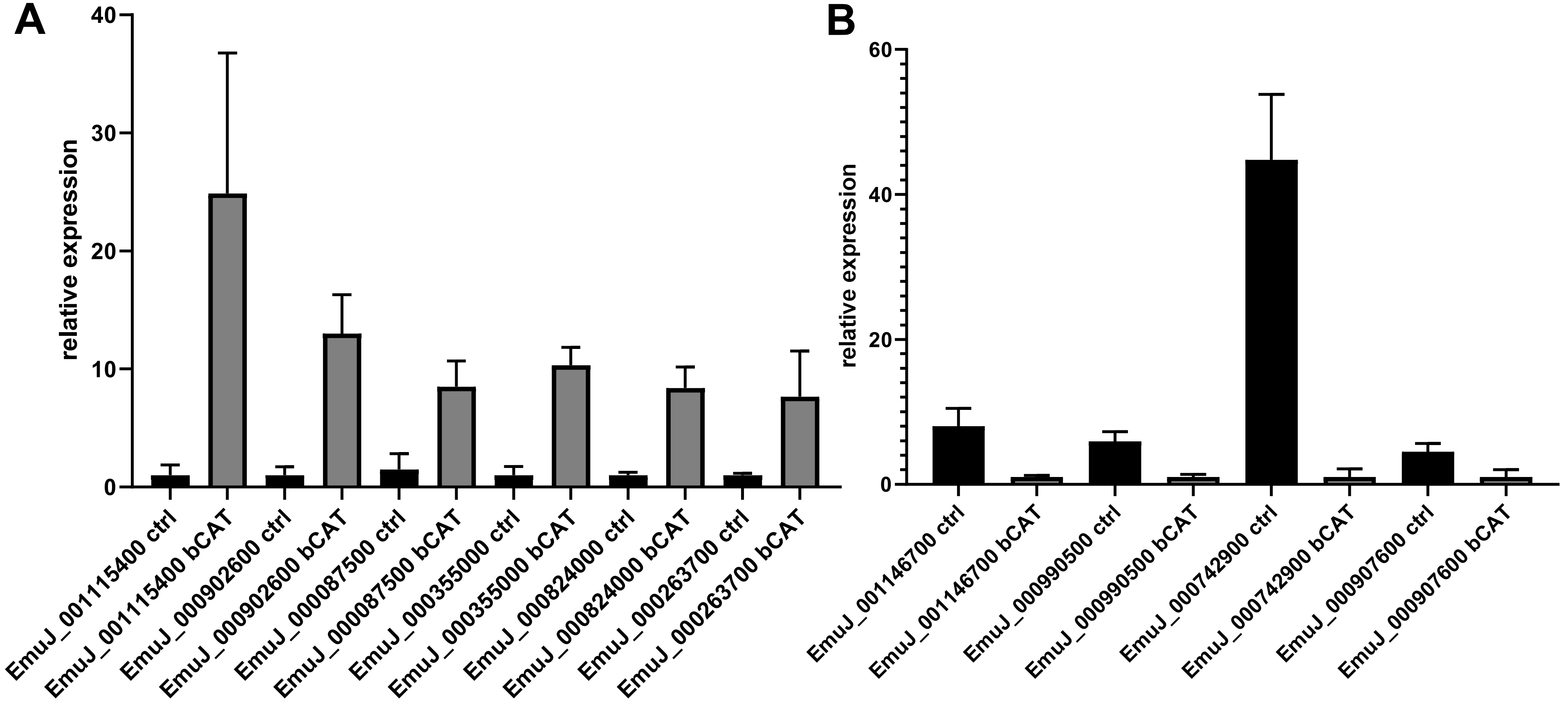

S6 Figure. qRT- PCR analysis of gene expression after siPOOL RNAi. A selection of genes that were significantly upregulated (A) or downregulated (B) after RNAi with siRNAs were analysed by qRT-PCR in cell cultures after siPOOL RNAi. Shown is relative expression in comparison to control gene *elp* (EmuJ_000485800). Indicated are gene IDs in control cultures (ctrl) and RNAi cultures (bCAT). Error bars indicate SD of three technical replicates.
