# Supplementary material for: Canonical WNT signalling governs Echinococcus metacestode development
Source: PLoS Pathog. 2026 Mar 23;22(3):e1014046. doi: 10.1371/journal.ppat.1014046 (PMC13029709; doi:10.1371/journal.ppat.1014046)
Supplement: S7 Fig — (PDF) [file ppat.1014046.s007.pdf]

S7 Figure

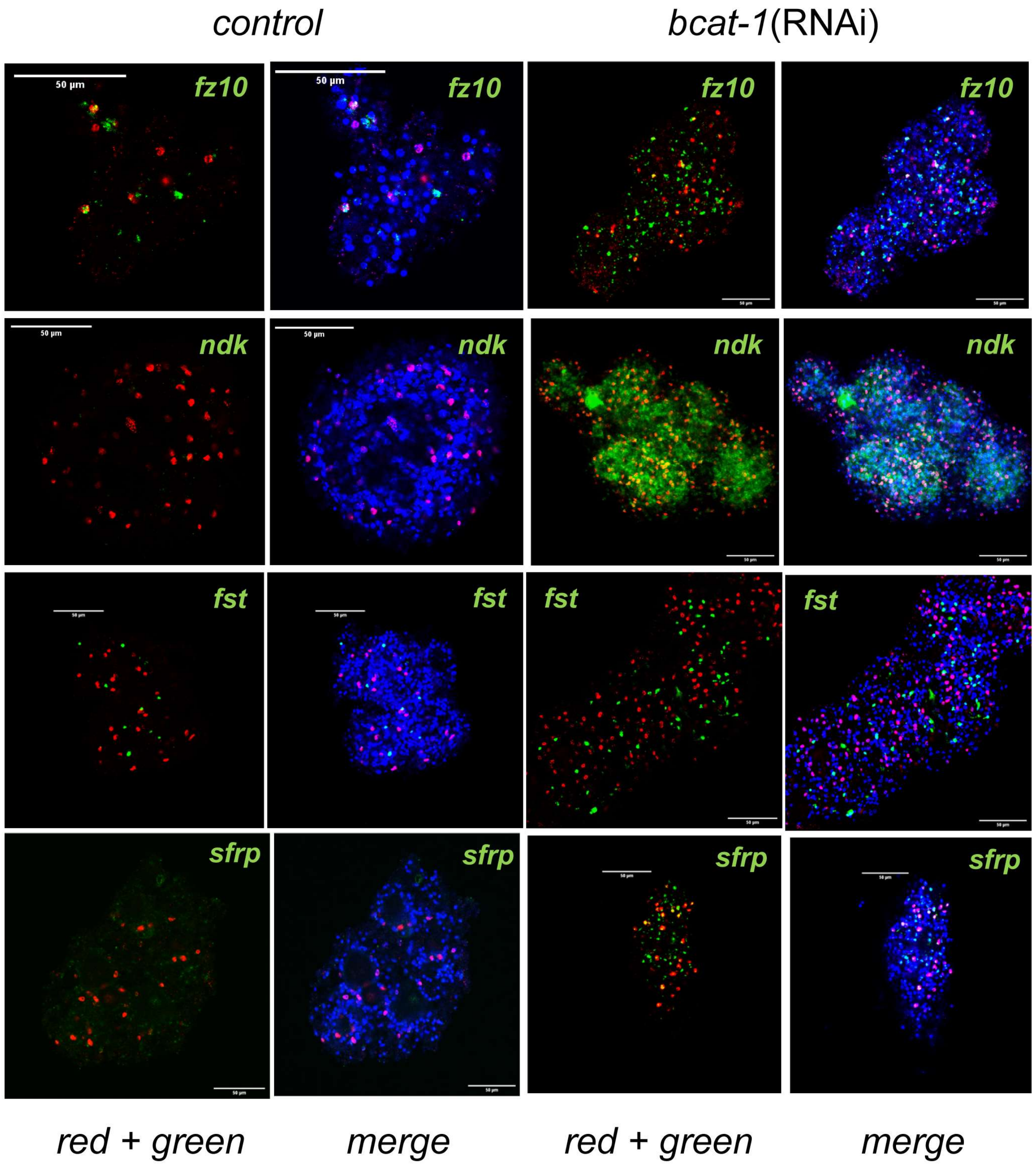

S7 Figure. Induction of anterior markers in *bcat-1*(RNAi) culture aggregates. WISH has been carried out on control RNAi cultures and bcat-1(RNAi) cultures (as indicated) for *fz10*, *ndk*, *fst*, and *sfrp* (from top to bottom). Shown are representative pictures for single confocal slices for red and green channel (red, Edu, proliferative stem cells; green, gene specific probe) and merge pictures also including third channel (blue, DAPI, nuclei) as indicated. Size bar represents 50 µm in all slides.
