# Supplementary material for: Canonical WNT signalling governs Echinococcus metacestode development
Source: PLoS Pathog. 2026 Mar 23;22(3):e1014046. doi: 10.1371/journal.ppat.1014046 (PMC13029709; doi:10.1371/journal.ppat.1014046)
Supplement: S8 Fig — (PDF) [file ppat.1014046.s008.pdf]

S8 Figure

A

Echinococcus multilocularis MUC2

```
MYFTQANDSASSDASAT                                                17

PTTRSTPKVTNPNLTTTGSTPTNPTTPKVTKPTVSTTASPPTSRTTPKVTKPTLTTTRAT     77

PTTRSTPKVTNPNLTTTGSSPTTRTTPQLTKPTATTTKATPTTRTTPKVTKPTLATTGST    137

PTTRTTPQLTKPTATTTSSTPTTQTTQITPKVTKPAATTTKATPTTRTTPKVTKPTDSTT    197

SSTPSNRTTPKLTKPTVSTTGSTTSTSTTPDISTPAATTTTATCPTCPSSSSSSPNTTAT    257

ETSAPIPPSICFWCLSPVWIAVIVVCVVLVCAVLVVICIVKFCCCRSDRAGRPGRGGQNS    317

NLLVNVNQV                                                       326
```

B

```
EmMUC2  18   PTTRSTPKVTNPNLTTTGSTPTNPTTPKVTKPTVSTTASPPTSRTTPKVTKPTLTTTRAT  77
             P TR  P  T P      S P     P  T P      S P +R  P  T P      +
HsMUC1  222  PDTRPAPGSTAPPAHGVTSAPDTRPAPGSTAPPAHGVTSAPDTRPAPGSTAPPAHGVTSA  281

EmMUC2  78   PTTRSTPKVTNPNLTTTGSSPTTRTTPQLTKPTATTTKATPTTRTTPKVTKPTLATTGST  137
             P TR  P  T P      S+P TR  P  T P A    + P TR  P  T P      S
HsMUC1  282  PDTRPAPGSTAPPAHGVTSAPDTRPAPGSTAPPAHGVTSAPDTRPAPGSTAPPAHGVTSA  341

EmMUC2  138  PTTRTTPQLTKPTATTTSSTPTTQTTQITPKVTKPAATTTKATPTTRTTPKVTKPTDSTT  197
             P TR  P  T P A   +S P T+     P  T P A    + P TR  P  T P
HsMUC1  342  PDTRPAPGSTAPPAHGVTSAPDTRP---APGSTAPPAHGVTSAPDTRPAPGSTAPPAHGV  398

EmMUC2  198  SSTPSNRTTPKLTKP  212
             +S P  R  P  T P
HsMUC1  399  TSAPDTRPAPGSTAP  413
```

S8 Figure. Structural features of *Echinococcus* MUC2. (A) Amino acid (aa) sequence of *E. multilocularis* MUC2 showing several highly similar, threonine-rich repeats between aa 18 and 257. Numbers to the right indicate MUC2 amino acids. A predicted transmembrane region is underlined. (B) Amino acid sequence comparison betwen repeat domains of MUC2 and human mucin MUC1 to which it shows highest homologies in SwissProt. Identical residues between both sequences are shown in interline. Biochemically similar residues are indicated by ‚+‘.
