# Supplementary material for: Canonical WNT signalling governs Echinococcus metacestode development
Source: PLoS Pathog. 2026 Mar 23;22(3):e1014046. doi: 10.1371/journal.ppat.1014046 (PMC13029709; doi:10.1371/journal.ppat.1014046)
Supplement: S9 Fig — (PDF) [file ppat.1014046.s009.pdf]

S9 Figure

A

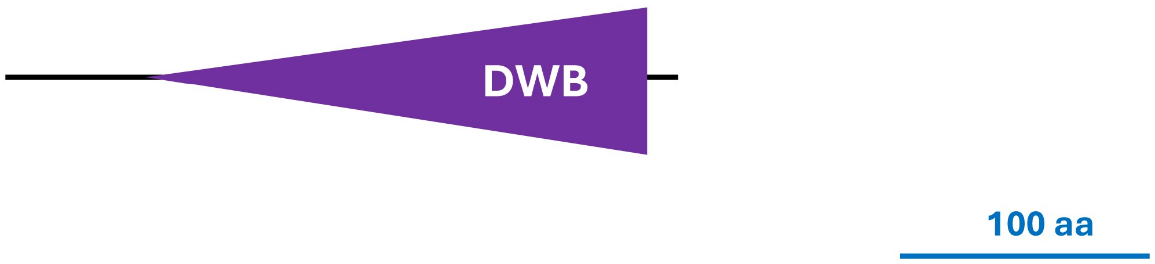

B

```
EmSmadF         KPWSHVSYWECNRHIGHKWLPVITSALVVLNEEHYSVSAATAENTSTHAVWSQQGRLCLD 60
SMAD6_HUMAN     SHWCSVAYWEHRTRVGRLYAVYDQAVSI-------------------FYDLPQGSGFCLG 41
SMAD7_HUMAN     SHWCVVAYWEEKTRVGRLYCVQEPSLDI-------------------FYDLPQGNGFCLG 41
                . *. *:*** . ::*: :     :  :                   .    * . :**.

EmSmadF         RLVRHFTSPRAFSEESHPSRKIYHPSILHSSSPTKQRRRLSHEGISLLLLPKGQILLTNK 120
SMAD6_HUMAN     QLNLEQRSE-------------------------SVRRTRSKIGFGILLSKEPDGVWAYN 76
SMAD7_HUMAN     QLNSDNKSQ-------------------------LVQKVRSKIGCGIQLTREVDGVWVYN 76
                :*  .  *                            ::  *: * .: *  : : : . :

EmSmadF         TLTTPIFVASPCFVQPGDLIAGDWPVYRVAPACSLVVFDTRIYED---RLTEAGKYTPWP 177
SMAD6_HUMAN     RGEHPIFVNSPTLDAPGGRA---LVVRKVPPGYSIKVFDFERSGL-QHAPEPDAADGPYD 132
SMAD7_HUMAN     RSSYPIFIKSATLDNPDSRT---LLVHKVFPGFSIKAFDYEKAYSLQRPNDHEFMQQPWT 133
                    ***: *  :  *..       * :* *. *: .** .                *:

EmSmadF         GKSLFGPVLHISLGKGWGPAYRRTDFTHCPARLEIWLN     215
SMAD6_HUMAN     -----PNSVRISFAKGWGPCYSRQFITSCPCWLEILLN     165
SMAD7_HUMAN     -----GFTVQISFVKGWGQCYTRQFISSCPCWLEVIFN     166
                        ::**: **** .* *  :: **. **: :*
```

S9 Figure. Structure and homologies of *Echinococcus* SmadF. (A) Domain structure of *E. multilocularis* SmadF. Shown is the characteristic DWB (dwarfin B) domain found at the C-terminus of Smads. Note that in contrast to human Smad6 and Smad7, an N-terminal DWA domain is missing in SmadF. Size bar indicates 100 amino acids. (B) Amino acid sequence comparison betwen DWB domains of *E. multilocularis S*madF, as well as human Smad6 and Smad7. Sittes of perfect alignment (*) as well as groups of strong (:) or weak (.) similarity are marked below the sequences.
