# Supplementary material for: Canonical WNT signalling governs Echinococcus metacestode development
Source: PLoS Pathog. 2026 Mar 23;22(3):e1014046. doi: 10.1371/journal.ppat.1014046 (PMC13029709; doi:10.1371/journal.ppat.1014046)
Supplement: S11 Fig — (PDF) [file ppat.1014046.s011.pdf]

S11 Figure

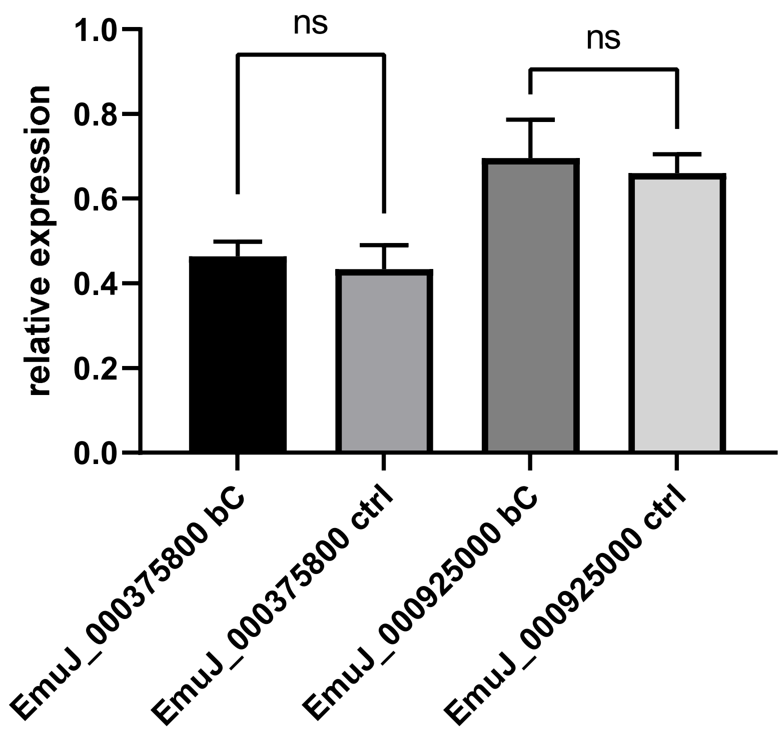

S11 Figure. qRT- PCR analysis of gene expression after siPOOL RNAi. To validate the control gene *elp* (EmuJ_000485800) as constitutively expressed factor upon bcat-1(RNAi), it‘s expression was measured by qRT-PCR in relation to genes EmuJ_000375800 and EmuJ_000925000. Error bars indicate SD of three technical replicates. Student‘s t-test. ns = not significant**.**
