# Supplementary material for: Canonical WNT signalling governs Echinococcus metacestode development
Source: PLoS Pathog. 2026 Mar 23;22(3):e1014046. doi: 10.1371/journal.ppat.1014046 (PMC13029709; doi:10.1371/journal.ppat.1014046)
Supplement: S10 Fig — (PDF) [file ppat.1014046.s010.pdf]

S10 Figure

A

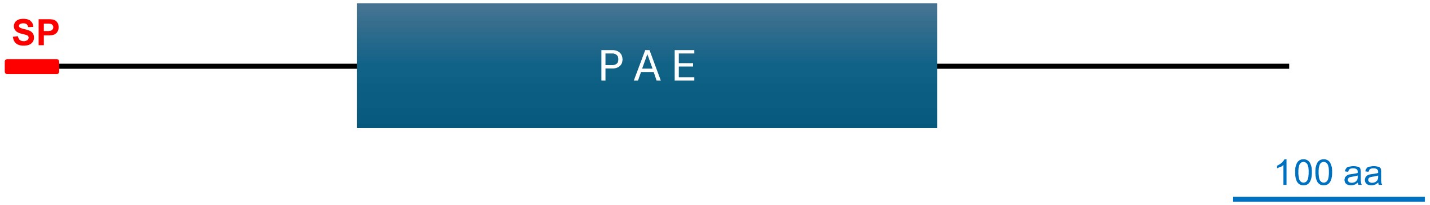

B

```
HsNOTUM          SAQQLNEDLRLHLLLNTSVTCNDGSPAGYYLKESR--GSRRWLLFLEGGWYCFNRENCDS      58
SCHMD_NOTUM      HSRKYQYFQLKKFPNSTNVRCNDGSIPGYYTRPSTTNCSKKWLIFLEGGWYCFNNNTCES      60
EmNOTUM          ------------------------HTDRYYVRRSKDLKSRNWLIYLEGGWYCFDEATCIS      36
                                             ** : *    *:.**::********:. .* *

HsNOTUM         RYDTMRRLMSSRDWPRTRTGTGILSSQPEENPYWWNANMVFIPYCSSDVWSGASSKSEKN       118
SCHMD_NOTUM     RRRTHYDLFSSEFWSSERQLGGILSNNERINPNFHDYNSVYIPYCSSDLWSGKQLE-KTN       119
EmNOTUM         RQITNHALFSSRTWHMIRYIGGVLSWDDRINPNYHNYNTIFVPYCSSDLWTGKKRE-RTG       95
                *  *   *:**. *   *   *:** : . ** : : * :::******:*:* . : ...

HsNOTUM         EYAFMGALIIQEVVRELLGR-GLSGAKVLLLAGSSAGGTGVLLNVDRVAEQLEK------       171
SCHMD_NOTUM     GLYFHGSRILDTVVDDLTQNQHFKKVHEVAFVGSSAGGIGVLLNIDRLKRRLKKKLKR--       177
EmNOTUM         GYYFHGSRILTAVIDDLLRQPEFIRVDKVVFSGSSAGGIGVMLNIDRLARRLRRGLRYRR       155
                   * *: *:  *: :*  .  :  .. : : ****** **:**:**: .:*.:

HsNOTUM         -----LGYPAIQVRGLADSGWFLDNKQYRH---TDCVDTITCAPTEAIRRGIRYWNGVVP       223
SCHMD_NOTUM     ---------KVFIHGIVDSAWFLDYPAYRQ---SNCTHIYECPPENALRNGMKLWNPRIP       225
EmNOTUM         WRQSTRNYSRLQISGLVDSAWFLHYPTYAQAYSGSCTSIYDCPPEEGILRGIGLWQPRIP       215
                          : : *:.**.***.   * :    .*.    * * :.: .*:  *:  :*

HsNOTUM         ERCRRQFQEGEEWNCFFGYKVYPTLRCPVFVVQWLFDEAQLTVDNVHLTGQPVQEGLRLY       283
SCHMD_NOTUM     RRCKKFQGRGREWKCFMGPVIYRHLKNPTFIIQSLFDDAQLQMSKVPILEGGSN-KKFSY       284
EmNOTUM         RRCRMAQGKKNFWQCYLGPVIYSHLRTPTFIIQSLFDEAQLQMSRAILLTGGSY-NKLAY       274
                .**:    . . *:*::*  :*  *: *.*::* ***:*** :... :           *

HsNOTUM         IQNLGRELRHTLKDVPASFAPACLSHEIIIRSHWTDVQVKGTSLPRALHCWDRSLH-          339
SCHMD_NOTUM     IQQLGGFAAQTLRQAKGVFAHSCVDHEILTKSNWAYVSVNNQRLHETLNYWQAYLEG          341
EmNOTUM         IQNLGKAVAKSLDTVQGVFAPACTDHEILTTNHWAQLMVDSQSLHDTLKAWDAHLT-          330
                **:**    ::*  . . ** :* .***:  .:*: : *..  *  :*: *:  *
```

S10 Figure. Structure and homologies of *Echinococcus* Notum. (A) Domain structure of *E. multilocularis* Notum. Shown are the characteristic palmitoleyl-protein carboxylesterase (PAE) domain (PAE) domain and the N-terminal signal peptide. Size bar indicates 100 amino acids. (B) Amino acid sequence comparison betwen PAE domains of Notum from *E. multilocularis* (EmNOTUM), human (HsNOTUM), and Schmidtea mediterranea (SCHMD_NOTUM) origin. Sittes of perfect alignment (*) as well as groups of strong (:) or weak (.) similarity are marked below the sequences.
