# Supplementary material for: Canonical WNT signalling governs Echinococcus metacestode development
Source: PLoS Pathog. 2026 Mar 23;22(3):e1014046. doi: 10.1371/journal.ppat.1014046 (PMC13029709; doi:10.1371/journal.ppat.1014046)
Supplement: Attachment — Submitted filename: Respone to reviews.docx [file ppat.1014046.s016.docx]

**Response to Reviewers**

Manuscript ID: PPATHOGENS-S-25-02863
Title: *Canonical WNT signalling governs Echinococcus metacestode development*

We thank the Editor and all three Reviewers for their careful evaluation of our manuscript and for their constructive comments. We appreciate the recognition of the methodological rigor of our study and the significance of establishing functional RNAi targeting β-catenin in *Echinococcus*.

We have carefully revised the manuscript in response to all comments. Editorial and formatting changes have been implemented throughout the manuscript and are summarized below. Importantly, we have addressed all conceptual and experimental concerns in detail, as outlined point-by-point below.

**Reviewer #1**

We thank Reviewer #1 for the positive evaluation of the novelty and relevance of our work and for the constructive suggestions.

**Rationale for experiments across developmental stages**

The reviewer suggested that the rationale for performing complementary experiments in different developmental stages should be explained more clearly.

We have revised the Introduction and Results sections to clarify that:

- RNAi-mediated knockdown is currently only feasible in primary cell cultures.
- Protoscoleces and metacestode vesicles are not amenable to robust RNAi approaches.
- Therefore, primary cultures were used for functional genetic perturbation of β-catenin, whereas in situ analyses were performed on intact larval stages to understand axis patterning in a developmental context.

We believe the revised text now makes the experimental logic transparent.

**Literature support and contextualization**

Where appropriate, we have strengthened the discussion with additional references, including recent studies on β-catenin knockdown phenotypes in *Hymenolepis diminuta* and *Fasciola hepatica*.

**Reviewer #1 – Comment:**
The reviewer asked about the relevance and rationale for using siPOOLs in addition to conventional siRNAs.

**Response:** siPOOLs consist of highly complex mixtures of independent siRNAs targeting the same transcript and are designed to minimize off-target effects while maintaining efficient knockdown. In our experiments, siPOOL-mediated knockdown of *bcat-1* resulted in phenotypes that were fully comparable to those observed with conventional siRNAs, including impaired vesicle formation, transcriptomic anteriorization, and increased stem cell proliferation. Importantly, in the revised manuscript we additionally show that siPOOL-treated cultures also exhibit stem cell hyperproliferation, demonstrating that the observed effects are not due to general toxicity but represent a specific consequence of β-catenin depletion. Moreover, we found that siPOOL-mediated knockdown was more efficient, as statistically significant effects on vesicle formation were already detectable after 3 days, whereas comparable effects were observed only after 7 days with conventional siRNAs. This increased efficiency likely reflects the optimized design of siPOOLs and further supports the robustness and specificity of the observed phenotype. We have clarified these points in the revised manuscript.

**Additional comments of reviewer #1:**

Reviewer #1: l. 46 – In situ should be italicised here and elsewhere in the manuscript.
1. 137 - In vitro should be italicised here and elsewhere in the manuscript.

**Response:** We corrected this.

Fig. 1, B and D – Please display/state level of significance.

**Response:** The significance level is now included.

l. 205 – Which group was used for staining, siRNAs or siPOOLs?

**Response:** We added the information throughout the manuscript.

l. 210 - 215 – The dysregulation of development and muscle organisation has also been reported following bcat knockdown in H. diminuta (Nanista et al., 2025) and F. hepatica (Armstrong et al., 2025) – it would seem most appropriate to reference these here.

**Response:** These references have now been included.

l. 230 – “These data indicate that the increased number of EdU⁺ cells reflect a general over-representation of stem cells in culture, not merely a short-term increased proliferation rate of otherwise constant numbers of stem cells.” – upregulation of 35.7% of stem cell-associated transcripts does not adequately support this statement. It would seem important to consider including additional evidence, e.g. EdU/BrdU pulse-chase in control vs bcat RNAi, or remove statement.

**Response:** The reviewer is right. We removed the statement.

l. 374 -379 – An image showing lack of expression of fz10 expression in a fully developed protoscolex, at least in supplementary info would be helpful.

**Response:** We included this image in the revised version. In addition, we also included images on fz10, ndk, and ndl-factors showing that they are not expressed in regular metacestode tissue.

Fig 4 – If s = suckers, please state this in legend.

**Response:** We did so in the revised version.

l. 433. Co-localisation with a muscle cell marker would be beneficial to support this statement.

**Response:** We meanwhile have produced single cell sequencing data showing that fst is indeed expressed in muscle cells. Those data are, however, not published yet. So, we removed the suggestion.

l. 618 - 619 – The generation of tegumental and hydatid-fluid components is multifaceted and disruption of the tegument following bcat RNAi was not evidenced in images – please adjust or remove statement to reduce speculation.

**Response:** In Echinococcus the tegument of the protoscolex differs from the tegument of the metacestode (e.g. there are much less microtriches in metacestode tegument and muc1 is specifically expressed in the metacestode tegument). What we wanted to express is that less metacestode-specific tegument is formed. So, the reviewer is right. We didn’t make this clear. We modified the statement in the revised version.

l. 656-669 – This section of text is highly speculative – please reduce.

**Response:** We removed the passage.

l. 680 – Using primary cell cultures for RNAi and protoscoloces for PP experiments diminishes the value of phenotypic comparisons – please explain rationale.

**Response:** Since we removed the PP section, this does no longer apply. However, to briefly explain the rationale: using RNAi we are currently only able to manipulate primary cell cultures. In protoscoleces, siRNAs at best only affect surface structures and numerous approaches to perform RNAi on metacestode vesicles failed so far. Hence, we attempted to cover those larval stages using a pharmacological approach – with pyrvinium being the most promising candidate at the moment. However, since we cannot presently be absolutely sure that pyrvinium targets beta-catenin, we decided to remove these data.

l. 687, 690, 700, 750, 766 – “essentially as previously described” – any deviations from the protocol referenced should be clearly stated.

**Response:** We apologize for this error. We did it ‘exactly’ as previously described and removed ‘essentially’ in the revised version.

**Reviewer #2**

We thank Reviewer #2 for the detailed critique and constructive suggestions, which improved the clarity of the manuscript.

**1. Visual introduction of developmental stages**

The reviewer correctly noted that the developmental stages of *Echinococcus* and the primary cell culture system were probably not sufficiently visualized, at least to the reader not familiar with the Echinococcus system.

We have therefore:

- Added two new supplementary figures illustrating:
  - The parasite life cycle from a developmental perspective.
  - The organization and developmental potential of the primary cell culture system.
- Integrated schematic representations into relevant figures to indicate:
  - Developmental stage
  - Tissue orientation
  - Cell types analyzed

We hope these additions substantially improve accessibility for readers unfamiliar with the system.

**2. Clarification of primary cell cultures**

We expanded the description of the primary cell culture system to clarify:

- Its enrichment in germinative stem cells.
- The presence of differentiated muscle and nerve cells.
- Its extended differentiation capacity, including formation of metacestode-like vesicles and cell types characteristic of other larval stages.

We also clarified similarities and differences to in vivo metacestodes in the Discussion.

**3. Pyrvinium pamoate experiments**

Reviewer #2 raised substantial concerns regarding specificity and interpretation of pyrvinium pamoate (PP) experiments.

We agree that:

- Direct evidence linking PP effects to β-catenin reduction in *Echinococcus* is currently lacking.
- Without a parasite-specific antibody against BCAT-1, we cannot conclusively demonstrate pathway-specific inhibition.

Therefore, we have **removed the entire PP section** from the manuscript.

We are convinced that the RNAi data alone provide conclusive and mechanistically clear evidence that canonical WNT/β-catenin signaling is instructive for axis formation and metacestode development.

**4. Transcriptomic anteriorization vs. vesicle phenotype**

The reviewer asked for clarification of the relationship between transcriptomic anteriorization and the red vesicle phenotype.

We have expanded the Discussion to explicitly link:

- Suppression of posterior/metacestode-specific genes (e.g., muc-1, Antigen B, TNFR, post2b)
- Upregulation of anterior patterning genes (sfrp, fz10, ndk, etc.)
- Failure to establish a posteriorized vesicle identity

We now argue that anteriorization prevents the acquisition of a posterior metacestode fate required for vesicle maturation.

**5. Increased proliferation after β-catenin inhibition**

The reviewer asked whether stem cell hyperproliferation was expected.

We now discuss two alternative interpretations:

1. Impaired differentiation toward posterior fates leading to accumulation of proliferating stem cells.
2. A shift toward an anterior developmental state, which physiologically correlates with increased proliferation during brood capsule and protoscolex formation.

We present these interpretations in a balanced manner and avoid overstatement.

**6. In vivo relevance of primary cell cultures**

We have expanded the Discussion to clarify:

- Which aspects of metacestode development are faithfully reproduced in vitro.
- Limitations due to absence of host-derived environmental cues.
- That primary cultures recapitulate intrinsic developmental programs but not the full host-parasite interface.

**Further comments of reviewer #2:**

-Figure 1 L-M, magnifications of EdU stained cells should be showed, to demonstrate the signal in the nucleus.

**Response:** We now provide magnified versions of the images. We also included a supplementary figure with all three channels showing that the signal is nuclear.

-Why is the “red-dot phenotype” obtained after beta-catenin inhibition? Showing magnifications and detailed explanations would help.

**Response:** We magnified the respective images in a revised figure. Since we already had observed the red-dot phenotype previously (our Ref 46) we already speculated about the red dots in this earlier work. The red colour most likely results from an accumulation of phenol red (as mentioned in our present work) since antigen B, a major component of hydatid fluid, has a high affinity to lipids. We also think that there are structural differences between the tegumental structures of ‘regular’ aggregates (reflecting the metacestode tegument) and bcat-1(RNAi) aggregates (reflecting more the protoscolex tegument), leading to an exclusion of phenol red in the former. Unfortunately, electron microscopic analyses which we had performed did not yield clear clues concerning the differences between both structures so that we presently can only rely on differences in gene expression (such as the expression of muc-1 in regular aggregates but not in bcat-1(RNAi) aggregates). We currently utilize additional markers for the metacestode tegument in WISH to obtain a clearer picture.

-In Figure 1, F-M, add labels to know what is control and RNAi. The same applies for figures 4-5-6-7. Add the concentration of the drug used, and the condition in each image. In Figure 5 add EdU in red in the images.

**Response:** We added the requested information.

-In Figure3, the magnification in Controls and RNAis of the same gene should be the same to be comparable.

**Response:** We prefer it the way we presented the images since we believe they are more informative. Size bars are included in all images so that the reader/reviewer gets a clear picture of dimensions. In some cases (e.g. for wnt11b, Post2, muc2) we showed an entire (somewhat larger) bcat-1(RNAi) aggregate to demonstrate lack of expression in the entire aggregate.

-Lines 210-212 ‘Moreover, muscle fibres in bcat-1(RNAi) aggregates were disorganized and only rarely accumulated around central cavities, a pattern typically observed during vesicle formation in this system (Fig 1J-M). The disorganization of muscle fibers is not evident. A magnification or an image of muscle fibers in the metacestode would help.

**Response:** An image of muscle fibres in the metacestode would not help here. Since primary cell aggregates are always somewhat disorganized when compared to a fully developed animal (e.g. a protoscolex) we even do not think that ‘disorganized’ is the right expression. We therefore changed the wording to ‘alteration’ of muscle fibres, since this is what we can clearly see.

-In Figure 1 J-K it seems that there are more neural cells in the RNAi than in the control. Authors could quantify it because it is very obvious in this image. If this is the case, it would fit with the anteriorized phenotype.

**Response:** Yes, it would fit. But it only appears so in this image. Since after inspection of all microscopic images we did not have the impression that the number of nerve cells significantly differs between control and bcat-1(RNAi) aggregates, we did not quantify this further. In contrast to counting EdU+ cells, counting nerve cells is much more arbitrary since we do not yet have a clear (genetic) nerve cell marker but just acetylated alpha-tubulin.

**Reviewer #3**

We thank Reviewer #3 for the thorough and very positive evaluation.

**WNT-independent roles of β-catenin**

The reviewer correctly pointed out that β-catenin also has WNT-independent roles (e.g., in adhesion and cytoskeletal organization).

We have added a clarifying paragraph in the Discussion acknowledging:

- Potential WNT-independent effects.
- Possible involvement of β-catenin in adhesion or cytoskeletal organization.
- The need for future work to disentangle these roles.

**qRT-PCR normalization and MIQE compliance**

The reviewer noted that only one reference gene (elp) was initially used.

We have now:

- Validated elp against two additional constitutively expressed genes.
- Included these data in a new supplementary figure (S11).

Additional comments of reviewer #3:

Line 177/178, Line 185: Homogenize decimals. Periods are used throughout the text and supporting tables, but some commas should be corrected: p < 0,0001

**Response:** corrected

Line 848 (n = 3) instead of (N = 3), like the others.

**Response:** corrected

S4 figure, italicize gene names fz10, ndk, fst, and sfrp. correct "indicaterd" in the legend.

**Response:** corrected

**Minor Editorial Issues**

All editorial suggestions from all reviewers have been implemented, including:

- Italicization of *in situ* and *in vitro*.
- Inclusion of statistical significance in figures.
- Clarification of siRNA vs. siPOOL conditions.
- Correction of decimal formatting.
- Clarification of tegument-related statements.
- Inclusion of magnified images where requested.
- Removal of speculative language where appropriate.

All changes are incorporated in the revised manuscript.

**Concluding Statement**

In summary:

- We removed the pharmacological section as suggested.
- We substantially improved visual guidance and developmental context.
- We expanded the Discussion to address anteriorization, hyperproliferation, and in vivo relevance.
- We strengthened normalization procedures and reporting transparency.

We believe the revised manuscript addresses all major and minor concerns and is now clearer, more focused, and methodologically robust.

We thank the Editor and Reviewers again for their constructive input and hope the revised manuscript will now be suitable for publication in *PLOS Pathogens*.
